# Molecular Characterization of Histamine-Producing Psychrotrophic Bacteria Isolated from Red Octopus (*Octopus maya*) in Refrigerated Storage

**DOI:** 10.3390/ht7030025

**Published:** 2018-09-04

**Authors:** Mariel Gullian Klanian, Mariana Delgadillo Díaz, Maria José Sánchez Solís

**Affiliations:** Experimental Unit, University Marist of Mérida, Periférico Norte Tablaje Catastral 13941, Carretera Mérida-Progreso, Mérida 97300, Yucatán, México; mdelgadillo@marista.edu.mx (M.D.D.); msanchez@marista.edu.mx (M.J.S.S.)

**Keywords:** *Octopus maya*, histamine-forming bacteria, decarboxylase genes, recombinant DNA

## Abstract

The present study aimed at determining the histamine production capacity of Gram (+) and Gram (−) bacteria isolated from *Octopus maya*, along with identifying the presence of amino acid decarboxylase genes. Of the total 80 psychrotrophic microorganisms, 32 strains were identified as histamine-forming bacteria. The recombinant DNA technique was used for genotypic identification of histidine (*hdc*), ornithine (*odc*), and lysine decarboxylases (*ldc*) genes. Thirty-two strains were able to produce 60–100 ppm in trypticase soy broth with 1.0% l-histidine after 6 h at 20 °C. NR6B showed 98% homology with *Hafnia alvei*. NR73 represented 18.8% of the total isolates and showed 98% homology with *Enterobacter xianfengensis* and *Enterobacter cloacae*. NR6A represented 6% of the total isolates, which were identified as *Lactococcus* sp. The *hdc* gen from NR6B showed 100% identity with *hdc* from *Morganella morganii*; *ldc* showed 97.7% identity with *ldc* from *Citrobacter freundii*. The *Odc* gene was detected only in NR73 and showed 100% identity with *Enterobacter* sp. All the isolated were identified as weak histamine–former. The ingestion of a food containing small amounts of histamine has little effect on humans; however, the formation of biogenic amines is often considered as an indicator of hygienic quality; this emphasizes the importance of improving good management practices and storage.

## 1. Introduction

Mexico ranks among the top 10 exporters of octopus to the United States, representing approximately 13% of the market. US octopus imports from Mexico have increased steadily, with almost 18,000 t imported in 2014. The fishery is mainly based on the red octopus *Octopus maya*, which is endemic to the Yucatan Peninsula, and a unique species among the 150-known species of octopus worldwide [1]. The extraction is done in a small-scale fleet with vessels between 8 and 10 m in length near the coastal areas (2–5-fathom depth) whereby the organisms have a high microbial load on the surface of the skin at the time of capture [2]. These daily trip-boats are not well equipped for cold storage, which renders the octopus a highly perishable product even in subsequent refrigerated storage.

Histamine (C_5_H_9_N_3_) has been identified as the main causative agent of scombrotoxic fish poisoning (often called “histamine poisoning”) which is caused by ingestion of certain species of marine fish that contain high levels of histamine and possibly other biogenic amines (BAs) [3]. The severity of the symptoms can vary, depending on the amount of histamine and other biogenic amines ingested and the individual’s sensitivity to specific BAs. Since fish handling practices are critical with regard to BAs production, histamine was defined as a chemical hazard regulated by the US Food and Drug Administration (FDA) according to the hazard analysis and critical control point (HACCP) guideline [4]. Failure to comply with these regulations and limitations leads to import rejections and disruptions in fish.

In particular, the presence of microbial populations with decarboxylase activity, the storage temperature and handling practices are the main factors affecting the production of BAs in raw seafood [5,6]. The illness associated with spoiled fish and fishery products is triggered by time/temperature abuse of fish muscle due to improper processing or storage [7,8,9,10]. A hazardous level of histamine (>50 mg/100 g) in muscle is often found with a prolonged storage even at the refrigeration temperatures [11,12]. Cadaverine (C_5_H_14_N_2_) and putrescine (C_4_H_12_N_2_) are known to enhance histamine toxicity by inhibiting histamine metabolizing enzymes such as diamine oxidase and methyl transferase, and they are also involved in nitrosamines formation, such as nitrosopiperidine and nitrosopyrrolidine, respectively [13,14,15].

The association of BAs with seafood safety has promoted empirical research for identifying all types of bacteria-producing decarboxylase enzymes, and such BAs are capable of forming histamine, cadaverine, and putrescine. Despite numerous reports showing that many bacterial species are capable of forming BAs, two groups of bacteria, the enteric and marine bacteria, have been identified as histamine formers in fish [16,17,18]. Both psychrophilic and mesophilic-halophilic histamine forming bacteria have been isolated from marine fish in the Gulf of Mexico [19,20]; however, the main concern related to seafood safety is the growth of psychrotrophic and psychrophilic histamine-forming bacteria (HFB), which can grow even at 0 °C. In particular, psychrotrophic microorganisms show a maximum growth temperature above 20 °C and are widespread in natural environments along with foods, while psychrophiles have a maximum temperature for growth at 20 °C or below and are restricted to permanently cold habitats [21]. 

Although there is a wealth of information about HFB in fishery products [22,23], they have been seldom studied in cephalopod and very little is known about BA production in octopus. Therefore, the objectives of this study were (a) to identify BA-forming bacteria in red octopus during refrigerated storage (4 ± 2 °C) and (b) to quantify the capacity of these microorganisms to produce histamine in the culture broth. 

## 2. Materials and Methods

### 2.1. Microorganisms

The microorganisms used in the present study were isolated earlier from the red octopus [24]. Of a total of 80 microorganisms isolated from the edible octopus’ tissues, three bacterial genera coded as NR6A, NR6B, and NR73 were found to be histamine producers. 

### 2.2. Isolation of Histamine Producing Bacteria

The isolated strains were cultured on Niven agar containing 0.5% tryptone, 0.5% yeast extract, 3% l-histidine, 0.5% NaCl, 0.1% CaCO_3_, 2% agar and 0.012% bromocresol purple; pH = 5.3 [25]. After 72 h incubating at 20 °C, the colonies were identified on the basis of the occurrence of a pH change in the agar adjacent to a growing colony (purple halo). Thus, 32 pure colonies were taken from the plate and sub-cultivated at 20 °C overnight in 5 mL of histidine broth containing 0.10% Bacto Peptone, 0.3% yeast extract, 0.5% glucose, 1.0% l-histidine, and 2% NaCl [26] and cryopreserved for further study. A strain of *Klebsiella pneumonia* isolated from fish and identified previously as a histamine former, was used as a positive control.

### 2.3. Phenotypic and Genotypic Identification of Histamine-Forming Bacteria 

The phenotypic identification was carried out using the commercial bacterial identification systems API 20E^®^ (Enterobacteriaceae and Gram-negative bacilli) (Biomerieux, Marcy-l’Étoile, France), API 20NE^®^ (Non-enteric Gram-negative bacilli) (Biomerieux, Marcy-l’Étoile, France), and complementary biochemical tests. Genomic DNA was obtained by lysis and protein digestion according to the method described by Ausubel et al. [27]. Briefly, 1.5 mL of overnight TSBH culture was centrifuged at 2600× *g* for 15 min at 4 °C (Eppendorf, 5804R, Westbury, NY, USA). Subsequently, the pellet was re-suspended in 600 μL of Tris-EDTA buffer containing 30 μL of 10% SDS and 3 μL of proteinase K (20 mg/mL). After 1 h of incubation at 37 °C, 100 μL of 5 M NaCl and 80 μL of 10% CTAB in 0.7 M NaCl were added and incubated at 65 °C for 10 min. The sample was washed twice, with chloroform/isoamyl alcohol 24:1 and with phenol/chloroform/isoamyl alcohol 25:24:1 and then centrifuged at 13,000× *g* each time to remove lipid and protein residues. DNA precipitation was achieved with 0.6 volumes of isopropanol. The DNA was washed with 300 μL of 70% ethanol and finally reconstituted in TE buffer (10 mM Tris-HCl, 1 mM Na-EDTA, pH 8.0).

Polymerase Chain Reaction (PCR) and subsequent sequencing of the 1400 bp region of the 16S ribosomal DNA (rDNA) was used for the genotypic identification. The universal primers 27F (5′AGA GTT TGA TCC TGG CTC AG 3′) and 1492R (5′GGT CT CTT GTT ACG ACT T 3′) were used for this purpose [26]. The PCR mixture reaction (25 μL) consisted of 10 mM Tris–HCL (pH 9.0), 50 mM KCl, 0.1% Triton X-100, 2.1 mM MgCl_2_, 0.2 μM of each deoxynucleoside triphosphate (dNTP), 0.4-μM primer concentration, 50 ng of bacterial DNA, and 1 U of Taq DNA polymerase (Promega Corp., Madison, WI, USA). The amplification profile was as follows: 1 cycle of 240 s at 94 °C, 40 cycles of 5 s at 94 °C, 45 s at 46 °C, and 90 s at 72 °C; and a final cycle of 10 min at 72 °C. PCR products were electrophoresed at 85 V in a 2% agarose gel. Amplicons were extracted from the gel and then purified and sequenced in an automated DNA sequencing instrument by the Sanger method (Applied Biosystems 3730xl; Thermo Fisher Scientific, Waltham, MA, USA).

The phylogenetic relationship of the strains was established considering on the statistical significance of matches of the representative 16S rDNA sequences retrieved from by the National Center for Biotechnology Information (NCBI) database (Bacteria and Archaea). The sequences were aligned with ClustalW using MEGA 7.0 software [28,29]. A phylogenetic tree was inferred using the Maximum Parsimony (MP) method with gaps deletion and bootstrapping of 500 replicates. MP tree was obtained by the random addition of sequences using the subtree pruning-regrafting algorithm [30].

### 2.4. Amplification of Histidine (Hdc) Ornithine (Odc) and Lysine Decarboxylase (Ldc) Genes 

The amplification of the target genes was carried out using oligonucleotides designed by De las Rivas et al. [31]. The *hdc* gene was amplified using the primer set 106 (5′AAY TCNTTYGAYTTYGARAARGARG3′) and 107 (5′ATN GGN GAN CCD ATC ATY TTR TGN CC 3′) that amplifies a region of 534 bp. The *odc* gene was amplified using PUT1-F (5′TWYMAYGCNGAYAARACNTAYYYTGT3′) and PUT1-R (5′ACRCANAGNACNCCNGGNGGRTANGG3′) amplifying a region of 1440 bp. Finally, the *ldc* gene was amplified with CAD1-F (5′TTYGAYWCNGCNTGGGTNCCNTAYAC3′) and CAD1-R (5′CCRTGDATRTCNGTYTCRAANCCNGG3′) with an amplification region of 1098 bp. The primer set 106/107 failed to amplify the *hdc* gene from the NR6A strain, which was then amplified by JV16HC (5′ AGA TGG TAT TGT TTC TTA TG 3′) and JV17HC (5′ AGA CCA TAC ACC ATA ACC TT 3′) primer set, specific for Gram-positive bacteria. This primer set generated a PCR product of 367-bp [32]. 

Amplification was performed in a 50 μL reaction mixture that contained 25 μL of DreamTaq Green PCR Master Mix (2X) (Thermo Scientific, Waltham, MA, USA) 75 pmol of each primer and 2 μg of DNA. The amplification was carried out for 35 cycles (95 °C for 30 s, 58 °C for 30 s and 72 °C for 1 min) in a Thermal Cycler (Major Science, Sea Gull Way Saratoga, CA, USA) with an initial denaturation of 94 °C for 4 min and a final extension at 72 °C for 7 min. The PCR products were separated on a 1.5% agarose gel at 80 V in 1X TAE buffer (400 mM Tris-acetate, 10 mM EDTA, pH = 8.2–8.4). The amplified fragments containing ethidium bromide (0.5 μg/mL) were visualized in a 3UV-Benchtop transilluminator (UVP, Upland, CA, USA). The product size was confirmed by comparison with a 100-bp molecular marker (Invitrogen, Carlsbad, CA, USA). The amplified products were purified using the Wizard SV Gel and PCR Clean-Up System (Promega Corp., Madison, WI, USA). Finally, the purified products were eluted and solubilized in nuclease-free water, quantified by fluorescence (Quantus Flurorometer E6150, Promega Corp., Madison, WI, USA) and stored at −20 °C for subsequent cloning and sequencing.

### 2.5. Cloning and Sequencing of Decarboxylase Genes

The amplicons from decarboxylase genes were treated with polyethylene glycol, cooled on the ice for 1 h, and pelleted by centrifugation at 15,000× *g* for 20 min. The pellet was washed with 70% ethanol, dried, and dissolved in Tris-EDTA buffer. The DNA fragments were ligated into a pGEM^®^ vector using the pGEM^®^-T Easy Vector System I ligation kit (Promega Corp., Madison, WI, USA) and transformed into *Escherichia coli* JM109 competent cells [33]. The ligation reaction was performed at 26 °C for 1 h and 4 °C for 48 h using 150 μL of competent cells. Heat shock was given by incubating the cells at 0 °C for 20 min, 42 °C for 45 s, and at 0 °C for 2 min. Subsequently, 1 mL of Luria-Bertani (LB) broth was added to each reaction and incubated at 37 °C with shaking. After 1 h, the cells were centrifuged at 5000× *g* for 15 min and re-suspended in 150 μL of LB broth. The transformed colonies were identified on LB agar plates containing ampicillin (100 μg/mL), 0.5 mM IPTG (isopropyl-β-d-thiogalactoside), and X-Gal (5-bromo-4-chloro-3-indolyl-β-d-galactopyranoside) (80 μg/mL) at 37 °C. After 18 h, the transformed cells with the insert (white colonies) and without the insert (blue colonies) were selected and cultured on LB agar plates with ampicillin (100 μg/mL) to extract the plasmid DNA.

### 2.6. Identification of Decarboxylase Genes in Transformed Cells

The identification of the cloned decarboxylase gene segments was carried out from the extraction of the plasmid DNA from the transformed cells by the alkaline lysis method [33]. Briefly, 1.5 mL of overnight TSBH culture of transformed cells was centrifuged at 2600× *g* for 3 min at 4 °C (Eppendorf, 5804R, Westbury, NY, USA). The pellet was then re-suspended in 100 μL of a cold solution containing 50 mM glucose, 25 mM Tris-Cl, 10 mM sodium EDTA at pH 8.0 and 200 μL of a solution consisting of 0.2 N NaOH and 1% SDS. After 3–5 min of cold rest, 150 μL of a cold solution containing 60 mL of 5 M potassium acetate, 11.5 mL of glacial acetic acid, and 28.5 mL of H_2_O were added. The sample was centrifuged at 2600× *g* for 10 min, and the pellet was washed with phenol/chloroform 1:1 (*v*/*v*). After centrifugation, the final pellet was washed with 100% and 70% ethanol, subsequently, and reconstituted with TE buffer containing 20 μg/mL of pancreatic RNAase.

The presence of the insert was verified using universal primers pUC/M13F (5′GTT TTC CCA GTC ACG AC 3′) and pUC/M13R (5′CAG GAA ACA GCT ATG AC 3′). The PCR amplification was performed in a 50 μL reaction mixture using 25 μL of the Dream Taq™ Green PCR Master Mix kit (2X), 1 μL of 2 pmol of each primer, and 2 μL of 1 μg of plasmid DNA sample. The amplification was carried out in 30 cycles (94 °C for 60 s, 55 °C for 60 s, and 72 °C for 2 min) with an initial denaturation at 94 °C for 5 min and a final extension at 72 °C for 10 min. The cloned DNA segments were sequenced by an automated DNA sequencer based on Sanger’s method (Applied Biosystems 3730xl; Thermo Fisher Scientific, Waltham, MA, USA). The deduced protein sequences were compared with UniProtKB database sequences (Swiss-Prot and TrEMBL). The sequences were aligned with ClustalW using MEGA 7.0 software [28,29]. The evolutionary relationship among the amino acid sequences was inferred by the Neighbor-Joining method [34]. The bootstrap consensus tree inferred from 500 replicates was taken to represent the relationships of the sequences analyzed [35]. The distances were computed using the Poisson’s correction method [36].

### 2.7. Determining of the Concentration of Histamine Produced by Bacteria

The concentration of histamine produced by the strains was determined by direct enzyme-linked immunosorbent assays (Multiskan EX, Thermo Scientific, Rockford, IL, USA) using the Veratox^®^ commercial kit. One colony was suspended in 50 mL of trypticase soy broth supplemented with 1.0% l-histidine (TSBH) and incubated at 20 °C in a shaking incubator at 150 rpm (Thermo Fisher Scientific, Waltham, MA, USA). At every hour, an aliquot of 3 mL was removed from the culture and used to estimate the total concentration of bacteria in soy trypcasein agar (TSA) and cell transmittance using a UV-visible spectrophotometer at 540 nm (GENESYSTM 10S, Thermo Fisher Scientific, Waltham, MA, USA). The remnant sample was centrifuged at 2600× *g* 15 min and the supernatant was used to quantify the histamine concentration.

## 3. Results

### 3.1. Molecular Identification of Histamine Producing Strains

Among the total 80 bacterial isolates from the muscle tissues, 32 strains were identified as histamine-producing by PCR. Table 1 shows the identity of the HFB determined by comparison of the sequences with NCBI-16S rDNA database. NR6B strain, which represented 15% of the total isolates, showed 98% homology with *Hafnia alvei* (NR_112985.1). NR73 represented 18.8% of the total isolates and 15 of them had 98% homology with *Enterobacter xiangfangensis* (NR_126208.1) and *Enterobacter cloacae* (NR_118568.1). Finally, the strain NR6A which represented 6% of the total isolates was identifying as *Lactococcus* sp. 

### 3.2. Phenotypic Characterization of Histamine Producing Strains

Of the 80 strains tested, 38 were identified as histamine forming by the Niven’s agar method but only 31 strains were positive by the molecular PCR-identification. With the exception of a *Lactococcus* sp. most of the histamine producing strains were Gram-negative. Two strains, viz., NR6B and NR73, from the *Enterobacteriacea* group, were characterized by catalyzing the decomposition of hydrogen peroxide to oxygen as well as the lack of cytochrome oxidase activity. Both strains were negative for the production of sodium thiosulfate, sodium pyruvate, and indole, and positive for glucose fermentation and nitrate reduction.

On the other hand, an immobile strain NR6A strain was catalase and oxidase negative and homofermentative, degrading glucose via glycolysis to produce lactic acid. Table 2 shows the complete phenotypic characterization of the HFB from red octopus.

### 3.3. Genotypic Identification of Histidine, Ornithine, and Lysine Decarboxylase Genes

**Figure 1:**
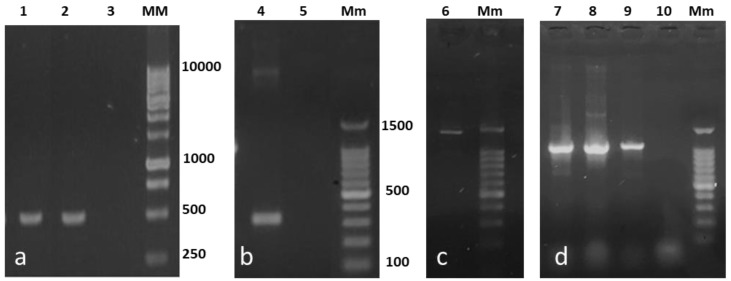
Identification of the histidine (*hdc*), ornithine (*odc*), and lysine (*ldc*) decarboxylase genes in the strains isolated from red octopus (*Octopus maya*). The *hdc* amplification of *Hafnia* *alvei* NR6B (1**a**) and *Enterobacter* NR73 (2**a**) was performed using the specific primer set 106/107 (534 bp); the primers JV16HC/JV17HC (367 bp) for *Lactococcus* NR6A (4**b**). *Odc* from *Enterobacter* NR73 (6**c**) was performed using the PUT1 primer set (1400 bp). *Ldc* from *H. alvei* NR6B (7**d**), *Enterobacter* NR73 (8**d**), and *Lactococcus* NR6A (9**d**) was amplified with the CAD1 primer set (1098 bp). Negative control = 3**a**, 5**b**, 10**d**; MM = 1 Kb DNA Ladder; Mm = DNA Ladder 100–1500 bp.

The detection of the decarboxylating genes by PCR confirmed the ability of the isolates to decarboxylate BAs and produce histamine, cadaverine, and putrescine. The 106/107 primer set (hdc gene) generated a typical PCR product of 534-bp for *H. alvei* NR6B and for *Enterobacter* NR73 (Figure 1a). This primer set failed to amplify the *hdc* in *Lactococcus* NR6A, which was later amplified by the JV16HC/JV17HC primers resulting in a 367-bp PCR product (Figure 1b). The *odc* gene was only detected in *Enterobacter* NR73 and generated a 1400-bp product (Figure 1c). The *ldc* gene was detected in all strains giving an amplicon of 1098 bp (Figure 1d). The results of the sequencing of decarboxylase enzymes are presented in Table 3. 

**Figure 2:**
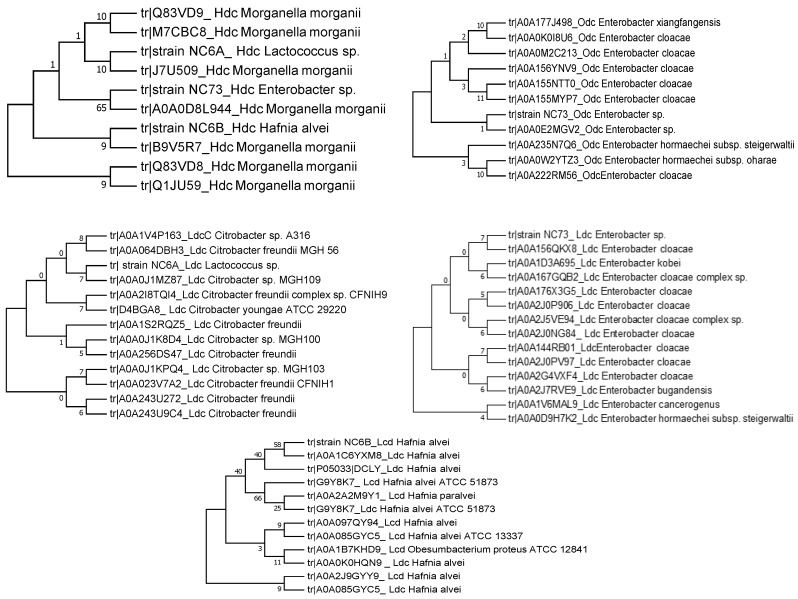
Evolutionary relationships of taxa of histidine (*Hdc*), ornithine (*Odc*), and lysine (*Ldc*) decarboxylase enzymes isolated from red octopus (*Octopus maya*) bacteria.

The *hdc* gene from *H. alvei* NR6B and *Lactococcus* NR6A presented 100% identity with the *hdc* sequence of *Morganella morganii*; *Enterobacter* NR74 showed 99.4% identity. For the *ldc* gene, *Enterobacter* NR74 showed 100% identity with *Enterobacter cloacae*; *H. alvei* NR6B presented 87% identity with the *ldc* gene from *H. alvei*, and *Lactococcus* NR6A had a 97.7% identity with *Citrobacter freundii*. The *Odc* gene was detected only in the *Enterobacter* NR74 showing 100% identity with the genus *Enterobacter*. The e-value, match length, and identity percentage for the referred alignment are shown in Table 4. The evolutionary relationship of the taxa based on the amino acid sequences of decarboxylase enzymes and inferred by the Neighbor-Joining method is shown in Figure 2.

The evolutionary history was inferred using the Neighbor-Joining method. The bootstrap consensus tree inferred from 500 replicates was taken to represent the evolutionary history of the taxa analyzed. The percentage of replicate trees in which the associated taxa clustered together in the bootstrap test (500 replicates) are shown next to the branches. 

### 3.4. Histamine Production

**Figure 3:**
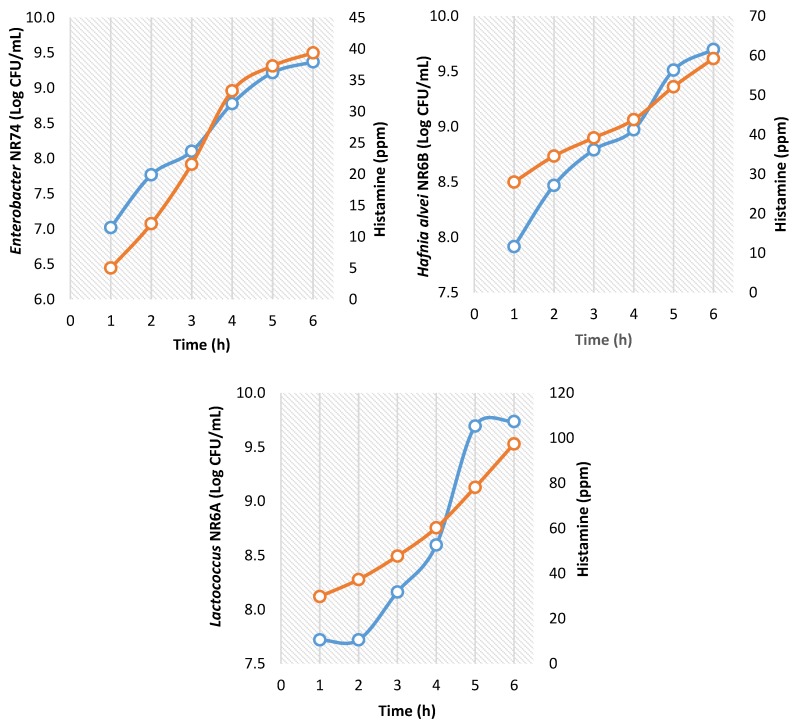
Bacterial concentration (blue) and TSBH-histamine production (orange) of the strains isolated from red octopus (*Octopus maya*); (The points correspond to an average of 3 replicates).

The concentration of histamine produced of the isolated strains in TSBH medium is shown in Figure 3. The histamine concentration was increased as a function of growth time. In the first six hours, the concentration of *H. alvei* NR6B, *Lactococcus* NR6A, and *Enterobacter* NR74 attained up to 9.0–10.5 log CFU/mL, but the production of histamine was variable. The highest histamine concentration was produced by *Lactococcus* NR6A reaching 100 ppm at 6 h of growth. *H. alvei* NR6B produced 60 ppm in the same time and *Enterobacter* NR74 reached less than 40 ppm in 6 h of growth. 

## 4. Discussion

Three bacterial strains isolated from the refrigerated octopus’ muscle were identified as BA-forming and were identified as *Hafnia*, *Enterobacter* sp. and *Lactococcus* sp. *Hafnia* was identified up to species level as *Hafnia alvei* (DNA group 1)*.*

There are only a few studies on the bacteria related to the production of BAs in octopus. Lougovois et al. [37] studied the spoilage potential of fresh musky octopus (*Eledone moschata*) by evaluating the changes in the biochemical properties, microbial growth, and sensory quality of the mantle and tentacles over a period of 18 days, and reported that the members of the *Enterobacteriaceae* were present only in small numbers. Other authors reported *Pseudomonas* spp., *Alteromonas putrefaciens*, *Flavobacterium* spp., *Shewanella* spp., *Acinetobacter* spp., and *Photobacerium* as prevalent bacterial species found in fish during cold storage; however, these genera have rarely been confirmed as HFB [26,38]. It is true that mesophilic *Enterobacteriaceae* exist as a minor bacterial flora in seafood stored at 0 °C, but psychrotolerant *Enterobacteriaceae* are capable of growing at refrigeration temperature and associate with the presence of BAs [26]. In the present study, 15 *Enterobacter* strains codified as NR73 were isolated from the muscle of red octopus and identified as HFB. Other *Enterobacter* species, e.g., *Enterobacter intermedium*, have previously been isolated from Bluefin tuna (*Thunnus thynnus*) at 8 °C confirming not only their adaptability to low temperatures but also the ability to produce histamine [39]. Identification at the species level in the genus *Enterobacter* is difficult on the basis of 16S rDNA, as the genus is polyphyletic [40]. The strain *Enterobacter* NR73 showed 98% identity with *Enterobacter xiangfangensis* sp. nov, and *Enterobacter cloacae* indicating a close relationship; however, the concatenated partial analysis based on the sequence of *rpoB*, *atpD*, *gyrB*, and *infB* genes should be analyzed in future studies to confirm a species [41].

*Hafnia alvei* was isolated from fresh and temperature-abused albacore (*Thunnus alalunga*) and found to be HFB by Kim et al. [16]. The genus *Hafnia* originally contained a single species, *H. alvei*, which was later described to include another species *H. paralvei* and some of the strains previously designated as the now obsolete *Obesumbacterium proteus*. *H. alvei* is genetically heterogeneous and consists of at least two DNA hybridization groups [42,43]. Recently, hybridization studies have distinguished the original *H. alvei* into *H. alvei* sensu stricto (DNA group 1) and *H. paralvei*, formerly known as *H. alvei* hybridization group 2 [44]. Based on the results of the biochemical tests carried out by Abbot et al. [45] for the identification of *Hafnia* species, the NC6B strain isolated in the present study was identified as *Hafnia alvei* (DNA group 1). *H. alvei* sensu stricto is typically malonate, salicin, and β-glucosidase positive, and d-arabinose negative, while *H. paralvei* has an opposite pattern. In our study, salicine, esculine, malonate, and β-glucosidase were typically positive and d-arabinose was negative.

Even knowing that decarboxylase is considered as species-specific or sometimes even strain specific activity, in the present study, *Hafnia alvei* NR6B and *Lactococcus* NR6A were identified as potential histamine and cadaverine producers, and *Enterobacter* NR73 as histamine, cadaverine, and putrescine producer. In fact, several studies have reported that the genera isolated from different foodstuffs show lysine and ornithine decarboxylase activity [46]. The *ldc* gene was found in the *Enterbocateriaceae* strains (*Hafnia* NR6B and *Enterobacter* NR73) amplifying a specific region of 1098-bp, but this region was also found in *Streptococcaceae* (*Lactococcus* NR6A). The primer set, CAD1-F, and CAD1-R, used in this study, were previously designed for some representative species of *Enterobacteriaceae* such as *Shigella flexneri*, *Shigella sonnei*, and *Escherichia coli* [31]. In the present study, this oligonucleotide set also amplified the *ldc* region from Gram-positive *Lactococcus* NR6A, which showed 97.7% identity with *ldc* from *Citrobacter freundii* (A0A0J1MZ87). The non-amplication of the *hdc* gene from *Lactococcus* NR6A with the primer set 106/107 was expected since the oligonucleotides were previously developed considering the two distinct classes of histidine decarboxylase enzymes; the homometric pyridoxal 5-phosphate (PLP)-dependent enzyme from Gram-negative bacteria [47], and the heterometric enzyme that contains an essential pyruvoyl group from Gram-positive bacteria [32,48].

It is difficult to predict the presence of cadaverine and putrescine in the octopus on the basis of gene expression. De Filippis et al. [49] mentioned that the expression of the decarboxylase genes, and the consequent production of cadaverine and putrescine in some microorganisms of the family *Enterobacteriaceae*, is influenced by the temperature, where a low temperature is associated with gene downregulation. In fact, the strains isolated from octopus, in particular, *H. alvei* NC6B and *Enterobacter* NC73 were found to be weak HFB compared with those reported by previous authors. For example, *Raoultella planticola* and *M. morganii* isolated from fish were identified as prolific histamine former with an ability to produce 1000 to 4000 ppm in 24 h at 37 °C in a TSBH medium [16,50,51].

The time and temperature of storage play a significant role in histamine accumulation. Torrido et al. [26] measured the histamine production of mesophilic and psychrotrophic isolates and concluded that, unlike the mesophilic, more than half of the 118 psychrophilic isolates produced no more than 500 ppm histamine. *H. alvei*, in particular, produced >88.7 ppm in fish infusion broth at 30 °C [52] but was identified as weak histamine former (42.1 ppm) in TSBH at 15 °C [53]. Kim et al. [38] also mentioned that only a few isolates of *H. alvei* and *E. cloacae* can produce >1000 ppm histamine in culture broth and the most of them are weak histamine formers. In our study, the histamine concentrations produced by the psychrotrophic *Enterobacter* NC73 and *H. alvei* NC6B after 6 h of incubation in TSBH were 40 and 60 ppm, respectively. This production classifies them as weak histamine forming strains.

Very little is known about the Gram-positive bacteria isolated from seafood and their role as HFB. The genus *Tetragenococcus*, created after reclassification of the halophilic lactic acid bacterium *Pediococcus halophilus* as *T. halophilus* is a Gram-positive microorganism isolated from salted and fermented fish products and was identified as an HFB [23,54]. A study reported the histamine production of 10.67 ppm from several strains of the *Lactobacillus* genus after 10 days at 25 °C [55]. This is relatively low compared to the 100 ppm produced for *Lactococcus* NC6A after 6 h in TSBH. The presence of the genus *Lactoccoccus* in a refrigerated octopus is unusual and possibly its presence is related to cross-contamination.

## 5. Conclusions

Although it is generally believed that histamine fish poisoning is caused by mesophilic histamine-producing bacteria that are active when temperature abuse occurs, this study showed that psychrophilic HFB are also prevalent in refrigerated octopus. To the best of our knowledge, this is the first report to demonstrate the occurrence of HFB in octopus; this emphasizes the importance of improving good management practices and storage. Although some of the enteric bacteria are naturally present in octopus’s tissues, most seem to arise from handling during harvesting and storage [24].

The isolated strains were found to produce a low level of histamine in the culture broth. The ingestion of food containing small amounts of histamine has little effect on humans; however, the formation of BAs is important not only from the standpoint of their toxicity, their presence is often being used as an indicator of hygienic quality and possible fecal contamination. Rapid chilling and refrigeration are mandatory to prevent microbial growth and consequent histamine formation. These are the main suggested control measures for seafood. In this sense, this study could assist in identifying better critical control points to minimize contamination of BAs-forming microorganisms in red octopus.

## Figures and Tables

**Table 1:** Identification of histamine-forming bacteria (HFB) from red octopus (*Octopus maya*) based on 16S rDNA sequences of pure isolates.

Code Strain	Closest Relative (Accession NCBI)	N^o^ Isolates	Total Score	Query Cover (%)	E-Value	Identity (%)
**NR73**	*Enterobacter xiangfangensis* (NR_126208.1)	15	1072	91	0.0	98
	*Enterobacter cloacae* (NR_118568.1)		1040	89	0.0	98
**NR6B**	*Obesumbacterium proteus* (NR_025334.1)	12	1652	98	0.0	98
	*Hafnia alvei* (NR_112985.1)		1646	98	0.0	98
	*Hafnia paralvei* (NR_116898.1)		1648	98	0.0	97
**NR6A**	*Lactococcus garvieae* (NR_113268.1)	5	1171	98	0.0	94
	*Lactococcus formosensis* (NR_114366.1)		1149	98	0.0	93

**Table 2:** Phenotypic identification of histamine-forming bacteria (HFB) isolated from the red octopus (*Octopus maya*).

Test Name	*Hafnia alvei* NR6B (*n* = 12)	*Enterobacter* NR73 (*n* = 15)	Test Name/*Lactococcus NR6A* (*n* = 5)			
Gram staining	−	−	Gram staining	+	Amygdaline	−
Motility	+	+	Motility	−	Arbutine	+
Catalase	+	+	Catalase	−	Esculine citrate	+
Cytochrome Oxidase	−	−	Cytochrome oxidase	−	Salicine	+
Histamine	+	+	Histamine	+	d-celobiose	+
ONPG production	+	+	Glycerol	−	d-maltose	+
l-Arginine-dihidrolase	−	+	Erythritol	−	d-lactose	+
l-Lysine decarboxilase	+	−	d-arabinose	−	d-melibiose	+
l-Ornithine decarboxilase	+	+	l-arabinose	−	d-saccharose	+
Trisodium citrate	+	+	d-ribose	−	d-trehalose	+
Sodium thiosulfate	−	−	d-xylose	+	Inuline	−
Sodium pyruvate	−	−	l-xylose	−	d-melezitose	−
Urea hydrolysis	−	+	d-adonitol	−	d-raffinose	+
l-Tryptophane	−	−	MD-xylopyranoside	−	Amidon	+
Indole production	−	−	d-galactose	−	Glycogene	−
Voges-Proskauer	−	+	d-glucose	+	Xylitol	−
Gelatine	−	−	d-fructose	−	Gentiobiose	−
Esculine ferric citrate	+	−	d-mannose	+	d-turanose	−
Capric acid	−	+/−	l-sorbose	−	d-lyxose	−
Adipic acid	−	−	l-rhamnose	−	d-tagatose	−
Malic acid	+	+	Dulcitol	−	d-fucose	−
Salicine	+	+	Potassium 5-cetoglutonate	−	d-arabitol	−
Malonate	+	+	Potassium 2-cetoglutonate	−	l-arabitol	−
β-glucosidase	+	+	Potassium gluconate	−	d-sorbitol	−
Acetyl glucosamine	+	+	M-ad-mannopyranoside	−	d-manitol	−
Potassium gluconate	+	+	Metyl-ad-glucopyranoside	−	Inositol	−
Nitrate reduction	+	+	*N*-acetylglucosamine	+	Malonate	−
d-Glucose fermentation	+	+	Gram staining	+		
d-Mannitol	+	+	Motility	−		
Inositol	−	−	Catalase	−		
d-Sorbitol	−	+/−	Cytochrome oxidase	−		
l-Rhamnose	+/−	+	Histamine	+		
d-Saccharose	−	+	Glycerol	−		
d-Melibiose	−	+	Erythritol	−		
Amygdaline	+/−	+/−	d-arabinose	−		
d-Arabinose	−	+	l-arabinose	−		
d-Mannose	+	+/−	d-ribose	−		
d-Maltose	+	+	d-xylose	+		

**Table 3:** Amino Acids Sequences of Decarboxylase Enzymes Isolated from Red Octopus (*Octopus maya*) Bacteria.

Code Strain	Enzyme	Amino Acid Sequence
**NR73**	*Hdc*	IPFEQSWGYVTNGGTEGNMFGCYLGREIFPDGTLYYSKDTHYSVAKIVKLLRIKSQVVESQPNGEIDYDDLMKKIADDKEAHPIIFANIGTTVRGAIDDIAEIQKRLKAAGIKREDYYLHADAALSGMILPFVDDAQPFTFADGIDSIGVSGHKMIGSPIP
	*Ldc*	GMSGERVPGKVFFETQSTHKMLAAFSQASLIHIKGEYDEDTFNEAFMMHTTTSPSYPLVASIETAAAMLRGNPGKRLINRSVERALHFRKEVQRLKDEADGWFFDIWQPEEIDEAECWPVAPGESWHGFRDADADHMF
	*Odc*	ALLTRGDLVLFDRNNHKSNHHGALIQAGATPVYLEAARNPFGFIGGIDEHCFDEAWLRELIRDVAPQKAAEARPFRLAIIQLGTYDGTIYNARQVIDKIGHLCDYILFDSAWVGYEQFIPMMAETSPLLLELNENDPGIFVTQSVHKQQAGFSQTSQIHK
**NR6B**	*Hdc*	FDFEKEVMEYFADLFKIPFEQSWGYVTNGGTEGNMFGCYLGREIFPDGTLYYSKDTHYSVAKIVKLLRIKSQVVESLPNGEIDYDDLMKKIADDKEAHPIIFANIGTTVRGAIDDIAEIQKRLKAAGIKREDYYLHADAALSGMILPFVDDAQPFTFADGID
	*Ldc*	CWPLDSKNPRNEWHGFPNIDNDHMYLDPIKVTLLTPGLSPNGTLEDEGIPASIVSKYLDEHGIIVEKTGPYNLLFLFSIGIDKTKALSLLRALTDFKRVYDLNLRVKNVLPSLYNEAPDFYKEMRIQELAQGIHALVKHHNLPDLMYRAFEVLPKLVMTPHDAFQEEVRGNIEPCALDDMLGKVSANMILPYPPGVPVVMPGEMLDTEEK
**NR6A**	*Hdc*	QSWGYVTNGGTEGNMFGCYLGREIFPDGTLYYSKDTHYSVAKIVKLLRIKSQVVESLPNGEIDYDDLMKKIADDKEAHPIIFANIGTTVRGAIDDIAEIQKRLKAAGIKREDYYLHADAALSGMILPFVDDAQPFTFADGIDSIGVSGHKMIGSPM
	*Ldc*	MSGERVPGKVIFETQSTJKMLAALSQASLIHIKGDNDEDTFNEAFMMHTSTSPSYPLVASIETAAAMLRGNSG

**Table 4:** Closest relative amino acids sequences of decarboxylase enzymes from strains isolated from red octopus (*Octopus maya*).

Code Strain	Enzyme	Closest Relative Amino Acid Sequences (Accession UniProtKB)	Query Length	Match Length	E-Value	Identity (%)
**NR73**	*Hdc*	*Morganella morganii* (*A0A0D8L944*)	510	378	4.9 × 10^−104^	99.4
	*Ldc*	*Enterobacter cloacae* (*A0A156QKX8*)	515	710	1.7 × 10^−^^101^	100
	*Odc*	*Enterobacter* sp. (*A0A0E2MGV2*)	433	711	2.9 × 10^−78^	100
**NR6B**	*Hdc*	*Morganella morganii* (*B9V5R7*)	513	378	4.9 × 10^−95^	100
	*Ldc*	*Hafnia alvei* (*A0A1C6YXM8*)	844	739	7.0 × 10^−147^	97.0
**NR6A**	*Hdc*	*Morganella morganii* (*J7U509*)	500	236	9.3 × 10^−92^	100
	*Ldc*	*Citrobacter freundii* (*A0A0J1MZ87*)	44	712	3.1 × 10^−101^	97.7
